# A School-Based Program for Problematic Internet Use for Adolescents in Japan

**DOI:** 10.3390/children10111754

**Published:** 2023-10-29

**Authors:** Yuichiro Otsuka, Yoshitaka Kaneita, Osamu Itani, Yuuki Matsumoto

**Affiliations:** 1 Division of Public Health, Department of Social Medicine, Nihon University School of Medicine, Itabashi-Ward, Tokyo 173-8610, Japan; otsuka.yuichiro@nihon-u.ac.jp (Y.O.); itani@iuhw.ac.jp (O.I.); yuuki_m@med.kurume-u.ac.jp (Y.M.); 2 Department of Public Health, International University of Health and Welfare, Chiba 286-8686, Japan; 3 Department of Nursing, School of Medicine, Kurume University School of Nursing, Kurume 830-0003, Japan

**Keywords:** adolescence, Internet addiction, population approach, transtheoretical model, educational measurement

## Abstract

Despite the serious influence of problematic Internet use on mental health among Japanese adolescents, no randomized clinical trials have investigated universal school-based interventions for this potential health challenge. Therefore, this study aimed to assess the efficacy of a school-based educational program. This two-armed, parallel, cluster-based randomized clinical trial included 5312 students from 13 high schools situated in a mid-sized Japanese city. The students in the intervention arm received 10 weekly standardized sessions, including a combination of information provision and interactive sessions by schoolteachers. The students in the control group followed a standard school curriculum. A generalized estimating equation model was applied to assess the primary (Korean Scale for Internet Addiction [K-scale] score) and secondary (behavioral change status according to changes in the transtheoretical model smartphone addiction score and Internet usage time) outcomes two months after intervention completion. The intention-to-treat analysis included 2597 (97.2%) and 2504 (94.9%) students in the intervention and control groups, respectively. Nevertheless, a significant discrepancy emerged regarding the behavioral change status. Therefore, this school-based program did not improve the Internet or smartphone addiction scores among Japanese adolescents. Further studies are needed to develop appropriate interventions for adolescents.

## 1. Introduction

The Internet is an indispensable tool in adolescents’ daily lives. However, problematic Internet use is recognized as a global public health concern [1]. Problematic Internet use has been defined as excessive Internet use that causes physical and psychosocial problems. It refers to various Internet-related behaviors, including web streaming, gaming, pornography viewing, bullying, gambling, social media use, and other behaviors [1]. Several reviews and meta-analyses have reported the negative effects of problematic Internet and smartphone use on mental health, academic performance, and overall quality of life [2,3,4,5]. In addition, adolescents beset by problematic Internet use frequently experience a decrease in physical activity [6]. A meta-analysis reported a problematic Internet use pooled prevalence of 4.6% (ranging from 0.6% to 19.9%) in adolescents, including 9.9% and 9.4% in Asia and North America, respectively [7]. Among Japanese adolescents, problematic Internet use is common, with the prevalence reaching 7.9% in nationwide representative surveys [8]. Similarly, a meta-analysis reported the median prevalence rate of problematic Internet use among children and adolescents to be 23.3% [5]. Thus, problematic Internet use is a significant public health concern among adolescents.

Problematic Internet use is particularly relevant to adolescents in Asia [5,9]. Adolescents surpass all other age cohorts in their utilization of the Internet [5], rendering them particularly susceptible to the health risks associated with problematic Internet use. Moreover, it is crucial to identify problematic Internet use during this formative phase, as research has indicated that addictive tendencies cultivated in adolescence have a proclivity to persist into adulthood [10]. Adolescents’ frequent engagement in potentially addictive online activities and their high frequency of online media use may be partially attributed to factors such as the fulfillment of needs [11,12], including peer communication [13], self-expression, and a desire for recognition, as well as the influence of peer group pressures [14]. A nationwide study among Japanese adolescents showed that prevalent activities associated with the risk of problematic Internet use risk include downloading (for both sexes); online gaming (for males); and social networking services, blogs, and bulletin boards (for females) [8]. A cohort study among Chinese adolescents demonstrated that online gaming use was a risk factor for problematic Internet use [15]. Furthermore, a cross-sectional study among Hong Kong adolescents revealed that recreational online endeavors, particularly engagement with social networking services (SNS) and online gaming, had a substantially higher proclivity for addiction than other online activities, including email correspondence and web browsing [16]. Gender disparities have been identified in Internet and smartphone utilization among adolescents. For instance, a cross-sectional study of Japanese adolescents revealed that females dedicated extended periods to activities such as online chat, SNS, and smartphone-based Internet browsing, while males allocated a greater portion of their time to gaming [17]. Similarly, a cross-sectional study involving UK adolescents reported that males spent an average of 3.25 h per day on gaming, while females spent 1.17 h [18]. Regarding social media engagement, males dedicated approximately 2.05 h per day, whereas females allocated 3.28 h. Additionally, Internet use per day totaled 3.79 h for males and 4.26 h for females [18]. These findings suggest that young men frequently engage in video gaming rather than utilizing smartphones.

Preventive interventions are crucial for mitigating problematic Internet and smartphone use. Although a recent meta-analysis demonstrated moderately favorable effects of short-term interventions on problematic Internet use, their overall significance was diminished owing to small sample sizes [19]. School-based prevention programs are a popular method applied in adolescents as they are cost-effective, have high participation rates, and cover the entire population at risk [20]. Exploring the effectiveness of school-based interventions for substance dependence, eating disorders, and gambling can enhance strategies for preventing problematic Internet use, as notable parallels exist between behavioral and non-behavioral dependence [21,22]. These may offer valuable insights into the prevention of problematic Internet use [23,24]. For example, school-based media literacy interventions for German adolescents showed a lower increase in Internet gaming frequency and time [25]. A recent systematic review has shown that school-based programs have a positive preventive effect on Internet addiction [20]. However, the varied nature of the available evidence prevents the formulation of overarching conclusions regarding their efficacy [20].

To date, no substantial randomized clinical trials concerning a universal school-based intervention for problematic Internet or smartphone use in Japan have been conducted. The development of scalable programs targeting adolescents with problematic Internet or smartphone use in resource-constrained environments is crucial and requires immediate attention. Japan is a high-income country with limited regulations. Thus, this cluster randomized clinical trial tested the effectiveness of a universal school-based program enforced by non-specialists to prevent problematic Internet or smartphone use among high school students in Japan.

## 2. Materials and Methods

### 2.1. Study Design 

This cluster randomized clinical trial adopted schools and students as the units of allocation and analysis, respectively, as concerns of potential cross-contamination among different classes within the same school emerged. The study was conducted from April to November 2021 (baseline survey: April to July 2021). After the baseline survey, teachers in the intervention group implemented the program. A follow-up survey was conducted 2 months after the intervention program or from September to November 2021 in the control group. All data were collected using self-administered questionnaires, and individual data from all assessments were linked while maintaining anonymity. The study received ethical approval and was registered with ClinicalTrials.gov under the identifier number UMIN000045062, conforming to CONSORT guidelines.

### 2.2. Setting, Participants, and Eligibility Criteria 

This study enrolled students (n = 12,285) in grades 10–12 from 21 high schools situated in a medium-sized Japanese city (population = 417,855 in 2021). The following procedures were used to identify and recruit volunteer high schools as participants:

(i) The contents of the trial were explained to the city’s school nurse teacher committee, and efforts were made to obtain consent for co-operation from schools;
(ii) A letter requesting an intervention study was sent to all school principals, and 13 principals agreed to participate.

Therefore, the target population was 13 schools with 5455 students in the 2021 academic year. 

The inclusion criteria for student participation were as follows:

(i) Students in 10–12 grades and their parents/guardians who agreed to participate in the study;
(ii) Students and their parents/guardians who willingly consented to participate after demonstrating sufficient understanding of the study following a detailed explanation of its aims and procedures;
(iii) Students and their parents/guardians who agreed to participate in the education program and could comply with the procedures related to the trial according to the instructions of the researcher or the teacher.

The exclusion criteria were as follows:

(i) Students who did not use the Internet;
(ii) Students and their parents/guardians who refused to participate in the program;
(iii) Students who were judged unsuitable for participation in the trial owing to health reasons cited by the school nurse.

**Figure 1:**
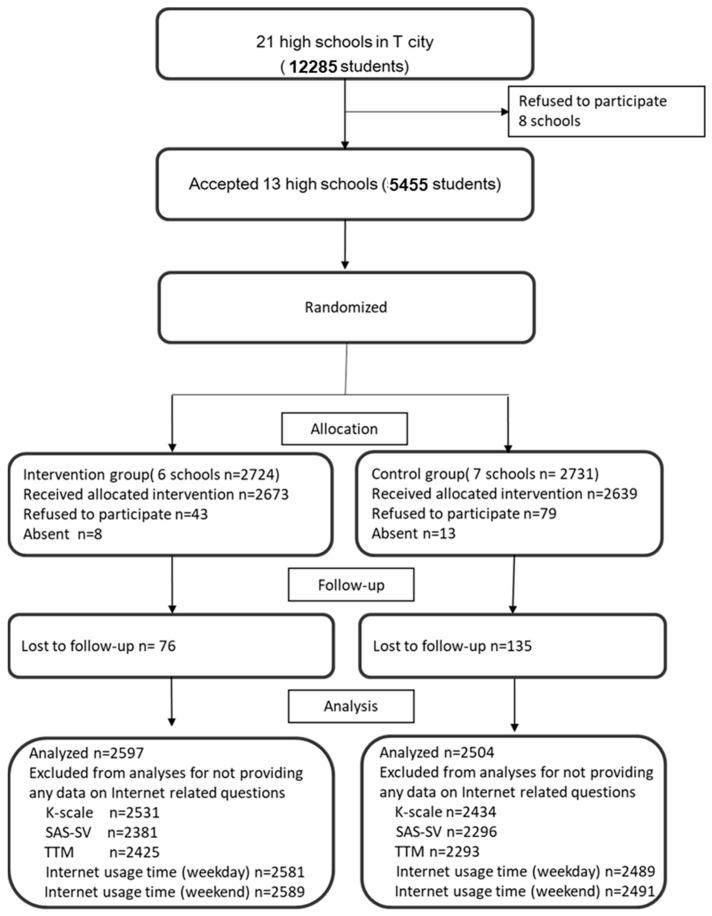
The CONSORT flow diagram. Flowchart of the school and adolescent recruitment process. K-scale, Korean Scale for Internet Addiction; SAS-SV, Smartphone Addiction Scale—short version; TTM, transtheoretical model.

Of all eligible students, 2.2% (n = 122) refused to participate, and 0.4% (n = 21) were absent on the day of the baseline survey. Participants provided written informed consent. The retention rate at follow-up was 97.4% (n = 5312). Therefore, of the 5312 students, 96.0% (n = 5101) were analyzed in the whole study (Figure 1).

### 2.3. Randomization 

The 13 schools were randomly assigned to the intervention (six schools) or control (seven schools) group before the baseline survey. Randomization aimed to balance the two groups regarding the number of classes in the participating schools, university advancement rates, and participating grades.

### 2.4. Intervention 

The intervention group adopted a hybrid approach comprising information provision and interactive educational modules delivered to the entire class during regular homeroom hours. It comprised 10 weekly sessions, each lasting approximately 10 min (Table 1). The first session was a self-reflection on students’ usage patterns to assess the Korean Scale for Internet Addiction (K-scale). From the second to third sessions, the teacher lectured on Internet addiction and related health problems. In the fourth and fifth sessions, students considered and discussed the advantages and disadvantages of Internet and smartphone use. In the sixth and seventh sessions, the students proposed problem-solving strategies. In the eighth session, the students recorded their Internet or smartphone use in diaries. In the ninth session, the students evaluated their usage patterns through these records. In the last session, the students revised their previous work and planned for future prevention. Each class or nursing teacher conducted the session. The materials provided to teachers encompassed reading materials and discussion guidelines to ensure consistency in the teaching approach. Students received workbooks containing key messages, discussion topics, and reviews of their behavior.

### 2.5. Control 

The control group received the standard curriculum during the survey period. After completing the intervention, they received the same curriculum as that of the intervention group. 

### 2.6. Outcome Measures 

The primary outcome measure was the K-scale [26], which provided a continuous score and was assessed 2 months after the intervention was completed. The 15-item short version of the K-scale is an assessment scale commonly used to screen for addictive Internet behaviors, particularly in Asian populations. The K-Scale demonstrates robust validity and reliability when employed as an assessment tool for evaluating addictive Internet behaviors among Japanese high school students [27]. The composite score represents the aggregation of responses to the 15 items on a four-point Likert scale, ranging from “strongly agree” (3 points) to “strongly disagree” (0 points). The high school students were categorized into three distinct Internet user groups as follows: Normal Internet users were defined as individuals who met either of the following conditions: (1) a total score of ≤40, or (2) scores ≤13, ≤11, and ≤11 for factors 1 (disturbance of adaptive functions), 3 (withdrawal), and 4 (tolerance), respectively. Potential problematic Internet users were identified as those with (1) a total score of 41–43 (inclusive), or (2) scores ≥14, ≥12, and ≥12 for factors 1, 3, and 4, respectively. Problematic Internet users were defined as individuals with (1) a total score of ≥44, or (2) scores ≥15, ≥13, and ≥14 for factors 1, 3, and 4, respectively [26]. The internal consistency test result (Cronbach’s alpha) was 0.844.

The secondary outcome measures were the changes in the transtheoretical model (TTM) stage [28], the short version of the smartphone addiction scale (SAS-SV) score [29], and Internet usage time. The TTM stages were categorized into five groups as follows: 1. precontemplation (not interested in the proper use of the Internet or smartphones), 2. contemplation (interested but unable to learn about the appropriate use of the Internet and smartphones), 3. preparation (preparing to implement the appropriate use of the Internet and smartphones), 4. action (<6 months of proper use of the Internet and smartphones), and 5. maintenance (>6 months of proper use of the Internet and smartphone and changes in processes). Changes in processes provide important guidance for intervention programs [28]. The SAS-SV was extracted from 10 questions from the original SAS for use as a screening tool in adolescents [29]. It is rated on a dimensional scale (1 = “strongly disagree” to 6 “strongly agree”). The total score ranges from 10 to 60, with the highest score representing an increased likelihood of smartphone addiction [29]. The internal consistency test result (Cronbach’s alpha) was 0.836. Self-reported Internet usage time was categorized into four levels as follows: <1 h, ≥1 h and <3 h, ≥3 h and <5 h, and ≥5 h. We defined excessive Internet use as ≥3 h and ≥5 h on weekdays and weekends, respectively [8,30]. All outcomes were assessed 2 months after the intervention was completed, as well as at baseline, using self-completed questionnaires. 

### 2.7. Covariates

Based on a previous study [4], the covariates included student age before the survey, sex, school, and depression. Depression was evaluated using the Patient Health Questionnaire 2. This scale showed good sensitivity and specificity for screening depression symptoms among adolescents [31]. The internal consistency test result (Cronbach’s alpha) was 0.634 in this study.

### 2.8. Sample Size 

The study aimed to include at least 1800 students in each group, amounting to a total sample size of 3600. This design allowed for a type I error of 0.05, a power of 0.80, 300 students per cluster, and an intraclass correlation coefficient of 0.05.

### 2.9. Statistical Analyses 

First, we employed descriptive statistics to assess the equilibrium at baseline in both groups. Second, to evaluate the effectiveness of the intervention, we fitted generalized estimating equation models with the school as a cluster variable to the following primary and secondary outcomes: K-scale score, TTM stage, SAS-SV score, and Internet usage time. The estimated standard errors were adjusted for clustering school, sex, grade, and depression using a Kauermann and Carroll bias-corrected variance estimator [32]. Finally, we conducted sensitivity analyses to assess the impact of missing data using multiple imputations. The results from both imputed and non-imputed data largely aligned, affirming that the primary findings were derived from two-level models exclusively relying on the observed data. All analyses adhered to the intention-to-treat principle. Statistical significance was set at *p* < 0.05. All analyses were conducted using Stata 17.0 (StataCorp LLC, College Station, TX, USA).

## 3. Results

Table 2 shows the baseline characteristics of the intervention and control groups. There were 49.7% and 46.7% males in the intervention and control groups, respectively. Approximately half of the students in both groups were in the 10th grade. The mean and standard deviations of K-scale scores were 33.7 ± 6.5 and 33.8 ± 6.5 for the intervention and control groups, respectively. Thus, the main outcome had similar distributions in the two arms. Notably, the intervention group demonstrated higher percentages for male students, lower SAS-SV scores, and longer Internet usage durations than the control group. 

Table 3 illustrates the primary and secondary measures. For the primary outcome, the K-scale scores showed no substantial distinction between the intervention and control groups (adjusted mean difference, 0.203; 95% confidence interval [CI], −0.516 to 0.922). Regarding the secondary endpoints, the intervention group demonstrated a minor favorable impact on the TTM stage (adjusted mean difference, 0.232; 95% CI, 0.072–0.392). Notably, no discernible evidence of differentiation was found between the groups for any other secondary criteria. Similar results were observed in the sensitivity analyses (Supplementary Table S1).

## 4. Discussion

To the best of our knowledge, this is the first large school-based cluster randomized study in Japan to assess a universal brief educational program to prevent problematic Internet and smartphone use. The intervention was meticulously designed based on existing evidence and achieved high levels of participant engagement. However, we observed no interventional effect on the Internet or smartphone addiction scores. The intervention only altered the stage of behavioral change. 

Despite large study heterogeneity, previous systematic reviews have shown positive preventive effects of school-based interventions on problematic Internet use [20,33]. For example, a cluster randomized controlled trial involving 2303 German adolescents documented a substantial influence of a media literacy program delivered by trained educators during standard class hours. Participants in the intervention group reported a decrease in the frequency and duration of gaming, along with a reduced proportion of excessive gamers compared to the control group [25]. In addition, another cluster-randomized controlled study employing cognitive-behavioral therapy-based intervention targeting 422 adolescents indicated that the intervention group had a significantly greater reduction in symptoms associated with gaming and unspecified Internet use disorders over 1 year when compared to the control group [34]. These results suggest that psychological interventions are effective for adolescents under certain conditions. 

Contrary to expectations, our findings showed that the classroom-based intervention lacked the intensity needed to achieve sufficient effects on problematic Internet or smartphone use. Several possible reasons may explain the ineffectiveness of this intervention. First, although the Internet plays an indispensable role in contemporary education and leisure, establishing ideal usage thresholds may present a paradoxical challenge, constituting a comprehensive and overarching concept that may necessitate additional contextual considerations. Given the Internet’s integral role in adolescents’ daily lives, the presumption underlying efforts to curtail Internet time as a preventive measure inherently implies that Internet usage is harmful to health. Second, the target population was not limited to those with high-risk Internet addiction. A population-level approach was chosen to provide support and assistance to students with subthreshold symptoms while also avoiding any potential stigmatization that may arise from specifically targeting individuals for intervention. This approach has limited advantages for individuals and results in poor motivation among participants [35]. Therefore, the effect of the intervention was expected to be minimal. Third, the duration of the intervention may have been relatively short or lacked appeal for students to engage in discussions regarding problematic Internet use. For example, a trial that recognized the effect of an intervention comprised four 90 min sessions [34]. Therefore, future programs should include longer interactive sessions aimed at changing attitudes and developing selected life skills [36]. Fourth, the efficacy of the intervention might have been diminished owing to the limited experience and training of the educators involved. However, this variability was relatively low to be estimated because we created instructional materials for the teachers. Thus, future studies should focus on enhancing the educational program’s quality by involving the expertise of psychologists, psychiatrists, and public health nurses well-versed in Internet-related issues to facilitate online classes. Alternatively, providing specialized training to teachers is an avenue for improvement. Finally, problematic Internet use is additionally linked to social variables, including parental and familial relationships [37]. A practical illustration involves a previously published randomized controlled trial on adolescents with Internet gaming disorder (IGD), which demonstrated a decrease in both the prevalence and symptoms of IGD through the application of family therapy [38]. Because of the high frequency of Internet use after school, it may be necessary to address not only students but also their families, peers, communities, and the environment.

This study has several strengths, including its cluster randomized controlled trial design, large sample size, high participant retention, and reliable measurements using the Internet/smartphone addiction scale. Moreover, the population approach may have facilitated behavioral changes. However, this study had some limitations. First, this study focused on students in the 10th to 12th grades from a specific provincial city in Japan. Therefore, it is imperative to approach the generalization of these results with caution, recognizing the potential for selection bias. Future investigations are underway to explore whether similar patterns emerge in different regions, thereby enhancing external validity. Second, we used self-administered questionnaires to assess the duration of Internet and smartphone usage. Measurements using objective devices, such as smart applications, to examine these variables may provide stronger evidence. Third, nonresponse and recall biases could have influenced the outcomes of our study. Despite our diligent efforts in providing prior clarifications and ensuring data privacy, a degree of bias may persist. Fourth, this study was conducted amidst the backdrop of the coronavirus disease 2019 (COVID-19) pandemic; as per a previous systematic review, alterations in daily routines attributable to COVID-19 may have influenced the manifestation of problematic Internet use [39]. Therefore, this may have reduced the efficacy of the intervention. 

## 5. Conclusions

The lack of substantial impact stemming from this school-based intervention for problematic Internet use underscores the need for program improvements. Schools represent an appealing setting for preventive or early treatment interventions that can assist adolescents in managing the vicissitudes of life. Understanding the mechanisms of problematic Internet use that underlie the specific active components of interventions is crucial. Therefore, further investigation is required to address the shortcomings in previous interventions and our interventions, thereby enabling the development and propagation of empirically substantiated interventions. For example, future educational interventions will necessitate an extended curriculum duration within educational institutions, in-depth and web-based sessions facilitated by skilled individuals, and interactive dialogues among students. Additionally, it is imperative to provide interventions that encompass not only the students but also their parents, peers, and the broader community.

Moreover, reaching a consensus regarding the terminology, clinical characterization, and evaluation of issues related to Internet overuse, gaming addiction, and social media addiction is essential to facilitate future comparisons among intervention studies. This, in turn, will enable the identification of critical components within these interventions. Such endeavors will contribute to the reinforcement of initiatives aimed at mitigating problematic Internet use, particularly among adolescents, potentially leading to the formulation of effective preventive strategies. Researchers and mental health practitioners increasingly acknowledge the imperative of devising and implementing preventative strategies, as they recognize that problematic Internet use poses a significant challenge for numerous adolescents. Policy makers and educational personnel must address this issue within two areas: public health and education policy.

## Figures and Tables

**Table 1:** In-school program for problematic Internet use for adolescents.

Session	Content
1	A self-reflection on students’ usage patterns to assess the Korean Scale for Internet Addiction (K-scale).
2	The teacher lectured on Internet or smatphone addiction.
3	The teacher lectured on health problems related to Internet or smartphone addiction and related behaviors.
4	The students considered the advantages and disadvantages of Internet and smartphone use.
5	The students discussed the advantages and disadvantages of Internet and smartphone use.
6	The students considered strategies to reduce their duration of digital device usage.
7	The students proposed problem-solving strategies that they could implement.
8	The students recorded their Internet or smartphone use in diaries.
9	The students evaluated their usage patterns through these records.
10	The students revised their previous work and planned for future prevention.

**Table 2:** Participant demographics and baseline characteristics.

	Intervention (N = 2673)		Control (N = 2639)		*p*-Value
Variable	N	%	N	%	
Sex					
Male	1328	49.7	1231	46.7	0.027
Female	1345	50.3	1408	53.4	
Grade					
10	1323	49.5	1302	49.3	0.313
11	1093	40.9	1051	39.8	
12	257	9.6	286	10.8	
Mental health					
Poor	1557	58.2	1550	58.7	0.896
Missing	142	5.3	112	4.2	
K-scale score	2586	33.7 ± 6.5	2540	33.8 ± 6.5	0.564
TTM stage					
Precontemplation	952	35.6	1153	43.7	<0.001
Contemplation	1096	41.0	997	37.8	
Preparation	213	8.0	162	6.1	
Action	118	4.4	95	3.6	
Maintenance	104	3.9	57	2.2	
Missing	190	7.1	175	6.6	
SAS-SV scores	2464	22.2 ± 7.3	2422	23.0 ± 7.8	<0.001
Prolonged Internet usage time (weekday)					
3 h+	913	34.6	1100	41.7	<0.001
Missing	26	1.0	19	0.7	
Prolonged Internet usage time (weekend)					
5 h+	897	34.0	969	36.7	0.001
Missing	19	0.7	15	0.6	

Data are presented as percentages or average ± standard deviation. *p*-values were calculated using chi-square tests for categorical variables or *t*-tests for continuous variables. K-scale: Korean Scale for Internet Addiction for adolescents; TTM: transtheoretical model; SAS-SV: Smartphone Addiction Scale—short version.

**Table 3:** Estimated mean differences in primary and secondary outcomes between the intervention and control groups.

Outcomes	n	Regression Coefficient (b)	95% CI		Df	t-Value	*p*-Value
Primary outcome							
K-scale score	4965	0.203	−0.516	0.922	8	0.65	0.533
Secondary outcomes							
SAS-SV score	4677	−0.386	−0.806	0.035	8	−2.12	0.067
TTM stage	4718	0.232	0.072	0.392	8	3.34	0.010
Excessive Internet usage time (weekday)	5070	−0.218	−0.605	0.169	8	−1.30	0.231
Excessive Internet usage time (weekday)	5080	−0.128	−0.362	0.106	8	−1.26	0.243

In each section, generalized estimating equation models were performed, and missing data were excluded from the statistical analyses. CI: confidence interval; K-scale: Korean Scale for Internet Addiction for adolescents; TTM: transtheoretical model; SAS-SV: Smartphone Addiction Scale—short version; Df: degree of freedom.

## Data Availability

The datasets generated and/or analyzed during the current study are not publicly available because the study involves human participants with a nondisclosure provision of individual data stated in the written informed consent in order to prevent compromise of study participants’ privacy but are available from the corresponding author upon reasonable request.

## Supplementary Materials

The following supporting information can be downloaded at: https://www.mdpi.com/article/10.3390/children10111754/s1, Table S1: Estimates of the mean difference in the primary and secondary outcomes between the intervention and control groups by generalized estimating equation models in the imputation data set.
