# Supplementary material for: A School-Based Program for Problematic Internet Use for Adolescents in Japan
Source: Children (Basel). 2023 Oct 29;10(11):1754. doi: 10.3390/children10111754 (PMC10670389; doi:10.3390/children10111754)
Supplement: Supplementary file 1 [file children-10-01754-s001.zip › children-2672360-supplementary.pdf]

Supplemental Table S1. Estimates of the mean difference in the primary and secondary outcomes between the intervention and control groups by generalized estimating equation models in the imputation data set

Outcomes	n	Regression coefficient (b)	95%CI			t-value	p-value
Primary outcome							
K-scale scores	5,111	0.168	-0.401	-	0.738	0.58	0.562
Secondary outcomes							
SAS-SV scores	5,111	-0.375	-0.673	-	-0.077	-2.47	0.014
TTM stage	5,111	0.207	0.066	-	0.347	2.88	0.004
Excessive internet usage (3 h+ weekday)	5,111	-0.500	-1.700	-	0.700	-0.82	0.414
Excessive internet usage (5 h+ weekend)	5,111	-0.263	-1.372	-	0.847	-0.46	0.643

CI, confidence interval

K-scale: The Korean Scale for Internet Addiction for adolescents

TTM: Transtheoretical model

SAS-SV: Smartphone Addiction Scale-short form version
